# miR-195 targets cyclin D3 and survivin to modulate the tumorigenesis of non-small cell lung cancer

**DOI:** 10.1038/s41419-017-0219-9

**Published:** 2018-02-07

**Authors:** Xiaojie Yu, Yiqiang Zhang, David Cavazos, Xiuye Ma, Zhenze Zhao, Liqin Du, Alexander Pertsemlidis

**Affiliations:** 1 Greehey Children’s Cancer Research Institute, The University of Texas Health Science Center at San Antonio, San Antonio, TX USA; 2 Department of Cell Systems and Anatomy, The University of Texas Health Science Center at San Antonio, San Antonio, TX USA; 3 Department of Chemistry and Biochemistry, Texas State University at San Marcos, San Marcos, TX USA; 4 Department of Pediatrics, The University of Texas Health Science Center at San Antonio, San Antonio, TX USA

## Abstract

miR-195 has recently been reported to function as a tumor suppressor in various cancers, including non-small cell lung cancer (NSCLC). However, the mechanisms by which miR-195 represses the tumorigenesis of NSCLC cells are not fully understood. We performed a high-throughput screen using an miRNA mimic library and confirmed the identification of miR-195 as a tumor suppressor in NSCLC. We demonstrated that overexpression or induced expression of miR-195 in lung tumors slows tumor growth and that repression of miR-195 accelerates tumor growth. In addition, we found that knockout of miR-195 promotes cancer cell growth. We demonstrated that miR-195 targets cyclin D3 to cause cell cycle arrest at the G1 phase and that miR-195 targets survivin to induce apoptosis and senescence in NSCLC cells. Overexpression of cyclin D3 or survivin reverses the effects of miR-195 in NSCLC cells. Through the analysis of data from The Cancer Genome Atlas, we confirmed that the expression of miR-195 is lower in tumors than in adjacent normal tissues and that low expression of miR-195 is associated with poor survival in both lung adenocarcinoma and squamous cell carcinoma patients. Specifically, we found that *BIRC5*, which codes for survivin, is upregulated in both adenocarcinoma and squamous cell carcinoma tissues and that high expression of *BIRC5* is associated with poor survival in adenocarcinoma, but not squamous cell carcinoma. In addition, the ratio of miR-195 level to *BIRC5* level is associated with both recurrence-free and overall survival in lung adenocarcinoma. Our results suggest that the miR-195/BIRC5 axis is a potential target for treatment of lung adenocarcinoma specifically, and NSCLC in general.

## Introduction

Lung cancer is the leading cause of cancer-related deaths worldwide^1^. Non-small cell lung cancer (NSCLC), including adenocarcinoma, squamous cell carcinoma, and large cell carcinoma, accounts for over 85% of lung cancers^2^. Studies have shown that microRNAs (miRNAs) play important roles in the initiation and progression of different cancers, including NSCLC^3^. Specifically, miR-195 has been reported to suppress cancer cell growth, migration, or invasion in different cancers^4–21^. The first indication of miR-195 relevance to NSCLC was its association with cellular response to drug treatment, based on the observation that miR-195 is upregulated in gemcitabine-resistant NSCLC cells^22^. The level of miR-195 in the plasma of patients has been proposed as a diagnostic and prognostic factor for NSCLC^23, 24^. Additionally, it has been shown that miR-195 expression can be used to classify lung adenocarcinoma into developing lung-like and adult lung-like subtypes, with the former demonstrating lower expression of miR-195 and worse overall survival^25^. These reports collectively suggest, but do not prove, that miR-195 plays significant roles in both the development of NSCLC and its response to chemotherapy.

Recently, it has been shown that miR-195 is downregulated in NSCLC tumor tissues and that increasing the level of miR-195 regulates cell cycle progression, migration, and invasion of NSCLC cells by targeting *MYB*^26^, *CHEK1*^27^, *HDGF*^8^, or *IGF1R*^13^. However, the specific mechanisms by which miR-195 represses NSCLC cell growth have not been completely elucidated. In this study, we demonstrate that repressing miR-195 activity promotes NSCLC growth, while increasing levels of miR-195 inhibits NSCLC growth. Specifically, we show that miR-195 regulates cell cycle progression, apoptosis, and senescence of NSCLC cells. We also identify *CCND3* and *BIRC5* as direct targets of miR-195 in NSCLC.

## Results

### miR-195 is a tumor suppressor in NSCLC

In order to identify miRNAs that repress the growth of NSCLC, we performed a high-throughput screen (HTS) in three NSCLC cell lines (NCI-H1155, NCI-H1993, and NCI-H358) and found that 74 miRNAs inhibit at least 25% of the average cell viability (Supplementary Table 1). Expecting to find tumor suppressor miRNAs downregulated in NSCLC, we analyzed miRNA expression in lung adenocarcinoma and squamous cell carcinoma patients from The Cancer Genome Atlas (TCGA, http://cancergenome.nih.gov). Forty-four miRNAs were found to be expressed at significantly lower levels in tumor tissues compared to adjacent normal tissues (Supplementary Table 2). Collectively, we found that only one miRNA (miR-195) both represses NSCLC cell growth and exhibits downregulation or lost expression in tumors relative to adjacent normal tissues (Table 1). Specifically, miR-195 is decreased in 83% (38 out of 46) lung adenocarcinoma patients and 96% (43 out of 45) squamous cell carcinoma patients, with lower expression of miR-195 associated with worse patient survival (Supplementary Figure 1A−C). Additionally, we compared miR-195 expression in NSCLC cell lines and several control cell lines (primary human bronchial epithelial cells (HBEpC), immortalized human bronchial epithelial cells (HBEC4-KT) and lung fibroblasts (WI-38 and IMR-90)). We could not establish whether or not miR-195 expression is strictly lower in NSCLC cell lines than in control cell lines due to the variance of miR-195 expression (Supplementary Figure 1D). However, we found that miR-195 is lower in NSCLC cancer cell line HCC4017 than in the immortalized normal lung epithelial cell line (HBEC30-KT) derived from the same patient (Supplementary Figure 7A).

**Table 1:** Candidate tumor suppressor miRNA(s) in NSCLC

	Viability					LUAD		LUSC	
miRNA	H358	H1155	H1993	Mean	*p*	T/N	*p*	T/N	*p*
miR-195	0.71	0.61	0.81	0.71	0.03	0.37	4.41×10^−13^	0.34	1.27×10^−14^

**Fig. 1:**
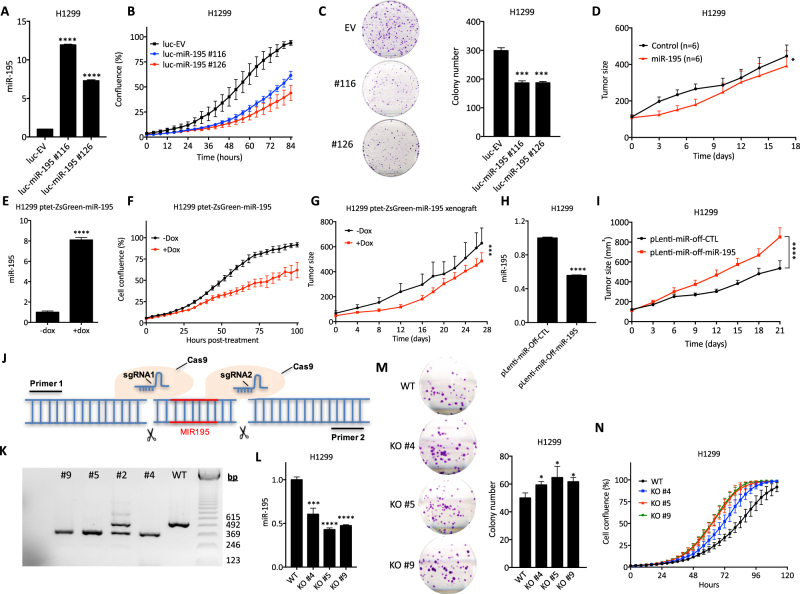
Activation of miR-195 is tumor suppressive, while repression of miR-195 is oncogenic. **a** Expression of miR-195 in control cells (H1299/luc-EV) and miR-195 overexpressing cells (H1299/luc-miR-195 #116 and #126). **b** Growth curves for control cells and miR-195 overexpressing cells. **c** Colony formation in control cells and miR-195 overexpressing cells. **d** Growth curves of tumors derived from H1299/luc-EV cells or H1299/luc-miR-195 #116 cells. **e** Doxycycline induction of miR-195 expression. **f** Doxycycline-induced miR-195 repression of cancer cell growth. **g** Growth curves of tumors derived from H1299/ptet-ZsGreen-miR-195 cells. When tumors reached 50−100 mm^3^, mice were given water containing 2 mg/mL doxycycline to induce miR-195 expression. The difference between tumor growth curves was assessed by two-way ANOVA. **h** miR-195 expression in control cells and miR-195 knockdown cells. **i** Growth curves of tumors derived from control cells and miR-195 knockdown cells. The difference between tumor growth curves was assessed by two-way ANOVA. **j** Schematic of the knockout of the *MIR195* locus. CRISPR/Cas9 was utilized to knock out a 147 bp DNA fragment containing *MIR195*, based on a pair of sgRNAs, one upstream of the *MIR195* locus and one downstream. **k** Agarose gel demonstrating the knockout of miR-195. In unmodified or wild-type (WT) H1299 cells, a pair of primers spanning the *MIR195* locus resulted in a 523 bp PCR product. In MIR195-knockout cells, only a 376 bp product was observed. DNA sequencing of the PCR products confirmed that the *MIR195* locus was knocked out in clones KO #4, #5, and #9. **l** miR-195 expression in wild-type (WT) cells and miR-195 knockout cells (KO #4, #5, and #9). **m** Colony formation assay of WT, KO #4, KO#5, and KO #9 cells. **n** Growth curves of WT, KO #4, KO#5, and KO #9 cells. *p < 0.05; ***p < 0.001; ****p < 0.0001

We validated the HTS results by demonstrating the dose−response of NSCLC cells to miR-195 mimic. However, we found that miR-195 also inhibits the growth of normal cell lines (Supplementary Figure 2A), indicating that the toxicity of miR-195 is not specific to cancer cells. To determine the effect of miR-195 on lung tumor growth, we established H1299 cells co-expressing miR-195 and luciferase (H1299/luc-miR-195). We observed that overexpression of miR-195 represses cancer cell growth *in vitro* and represses tumor growth *in vivo* (Fig. 1a–d, Supplementary Figure 4A). In addition, we established miR-195-inducible H1299 cells in which doxycycline treatment induces co-expression of miR-195 with ZsGreen fluorescent protein (Fig. 1e, Supplementary Figure 3A, B). We observed that induction of miR-195 inhibits cancer cell growth both *in vitro* and i*n vivo* (Fig. 1f, g, Supplementary Figure 4B). We also used lentivirus to co-express miR-195 inhibitor with GFP (Supplementary Figure 3C, D). In miR-195 inhibitor-overexpressing H1299 cells (H1299/pLenti-miR-Off-miR-195), the level of miR-195 is significantly reduced, which accelerates lung tumor growth (Fig. 1h, i). Similarly, knockout of miR-195 using CRISPR-Cas9 promotes cancer cell growth (Fig. 1j–n). The lower expression of miR-195 in NSCLC tumors, the correlation of miR-195 levels with patient survival, and the ability of miR-195 to regulate NSCLC growth strongly suggest that miR-195 acts as a tumor suppressor in NSCLC.

### miR-195 delays cell cycle progression and induces apoptosis and senescence in NSCLC

**Fig. 2:**
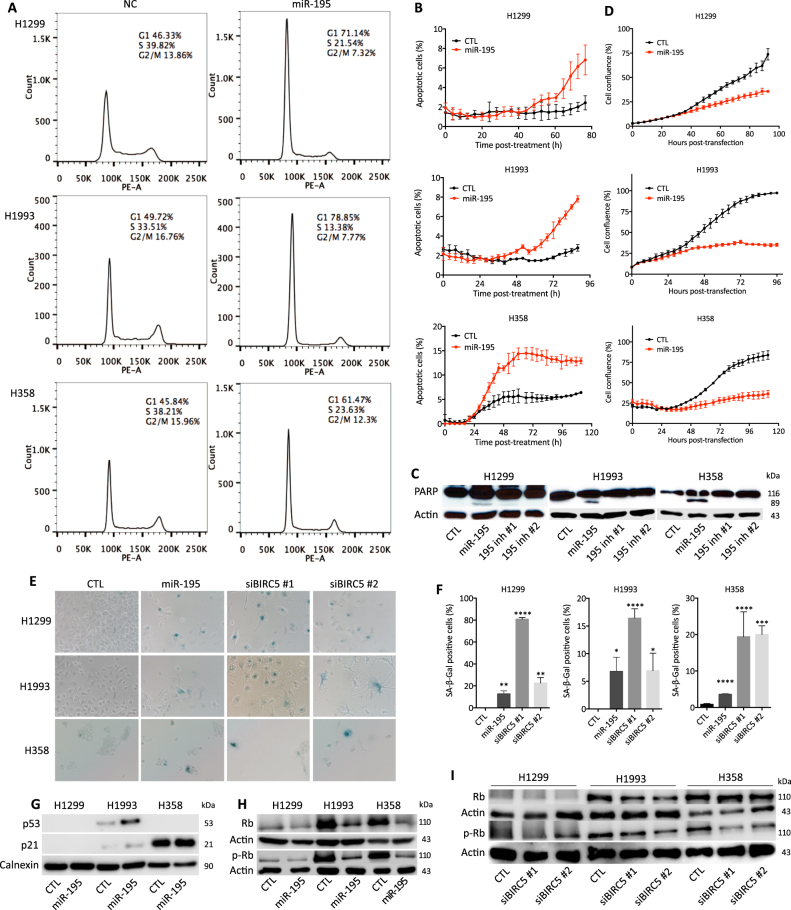
miR-195 delays cell cycle progression at G1 and induces apoptosis and senescence in NSCLC cells. **a** Cell cycle distribution as a function of miR-195 transfection in NSCLC cells. Fractions of cells in G1, S, and G2 phases were estimated using the Watson pragmatic model. **b** Time-dependence of induction of apoptosis by miR-195 in NSCLC cells. **c** PARP and cleaved PARP protein levels in NSCLC cells transfected with miR-195 mimic and inhibitors. **d** Cell confluence as a function of time after miR-195 transfection in NSCLC cells, quantified using the IncuCyte FLR live cell imaging system. **e** β-gal staining of cells transfected with miR-195 mimic or BIRC5 siRNAs. **f** Quantification of β-gal staining. **g** p53 and p21 protein levels in NSCLC cells transfected with control or miR-195 oligos. **h** Rb and p-Rb protein levels in NSCLC cells transfected with control or miR-195 oligos. **i** Rb and p-Rb protein levels in NSCLC cells after BIRC5 knockdown. *p < 0.05; **p < 0.01; ***p < 0.001; ****p < 0.0001

In order to elucidate the mechanisms by which miR-195 inhibits NSCLC cell growth, we examined the change in cell cycle distribution in response to miR-195 transfection. We observed significantly more cells in the G1 phase after miR-195 transfection (Fig. 2a), suggesting that miR-195 causes arrest of NSCLC cells in the G1 phase. Additionally, we detected apoptotic cells induced by miR-195 through caspase-3 activation measured by live cell imaging and PARP cleavage assessed by western blot (Fig. 2b, c).

Cellular senescence, growth arrest that limits cell proliferation, is also important for tumor suppression. We found that miR-195 significantly increases the number of senescent cells, as measured by β-galactosidase staining assay (Fig. 2e, f). Since p16, the most commonly used marker for senescence, is frequently deleted in lung cancer^28, 29^, we did not detect any p16 protein. We found that p53 protein is increased by miR-195 in H1993 cells, but not in H1299 and H358 cells, where it is deleted (Fig. 2g). We also found decreased levels of p-Rb by miR-195, indicating both activation of the retinoblastoma (Rb) pathway and senescence (Fig. 2h).

miR-195 has also been shown to cause mitochondrial dysfunction in breast cancer cells^30^. We evaluated the effect of miR-195 on mitochondrial respiration in NSCLC cells. A549 cells were chosen because relevant assays have been optimized for those cells. We did not find the oxygen consumption rate and extracellular acidification rate to be significantly different between miR-195- and control-treated cells, indicating that miR-195 does not regulate mitochondrial respiration in A549 cells (Supplementary Figure 5).

### miR-195 directly targets *CCND3* and *BIRC5* to inhibit NSCLC

To identify the genes directly targeted by miR-195 in NSCLC, expression profiling was performed in two NSCLC cell lines (H358 and H1993) following transient transfection with miR-195. Direct targets of miR-195 reported in NSCLC, such as *MYB*, *CHEK1*, and *HDGF*, but not *IGF1R*, were observed to be downregulated by miR-195 (Supplementary Table 3). Several targets of miR-195 reported in other cancers, such as *VEGF* and *BTRC*, are not decreased by miR-195 in NSCLC, supporting the idea that the target space of a given miRNA is context-dependent. Analysis of downregulated genes using Ingenuity Pathway Analysis identified canonical pathways that are regulated by miR-195, mostly related to cell cycle regulation and DNA damage response, supporting our findings that miR-195 induces cell cycle arrest, apoptosis, and senescence in NSCLC cells (Supplementary Tables 4, 5).

We then examined cancer-related genes harboring the target region of miR-195 (“TGCTGCT” sequence) in their 3′UTRs and found that the expression of ten genes decreased by more than twofold (Table 2). Among the ten genes, the functions of *CCND3* and *BIRC5* can explain the roles of miR-195 in NSCLC cells: *CCND3* encodes for cyclin D3 and is responsible for regulation of G1/S transition in cell cycle^31^; *BIRC5* encodes survivin, which is a small inhibitor of apoptosis (IAP)^32^, and regulates senescence, migration, and invasion of cancer cells^33, 34^. Therefore, we anticipated that *CCND3* and *BIRC5* are direct targets of miR-195 in NSCLC.

**Table 2:** Candidate target genes of miR-195 in NSCLC

Gene	H358	H1993	Pathways
*CCND3*	−2.54	−3.35	Cell cycle: G1/S
*BIRC5*	−2.90	−2.76	Apoptosis, migration, cell cycle: G2/M
*ANLN*	−2.77	−2.17	Cell growth, migration
*NAE1*	−2.31	−2.10	Cell cycle: S/M
*TACC1*	−2.13	−2.14	Breast cancer
*CHEK1*	−2.30	−2.00	Cell cycle: G2/M
*CARD10*	−2.08	−2.05	Apoptosis
*ARL2*	−4.09	−4.81	Apoptosis
*SPRYD3*	−2.39	−3.38	NA
*PCMT1*	−2.45	−2.55	NA

**Fig. 3:**
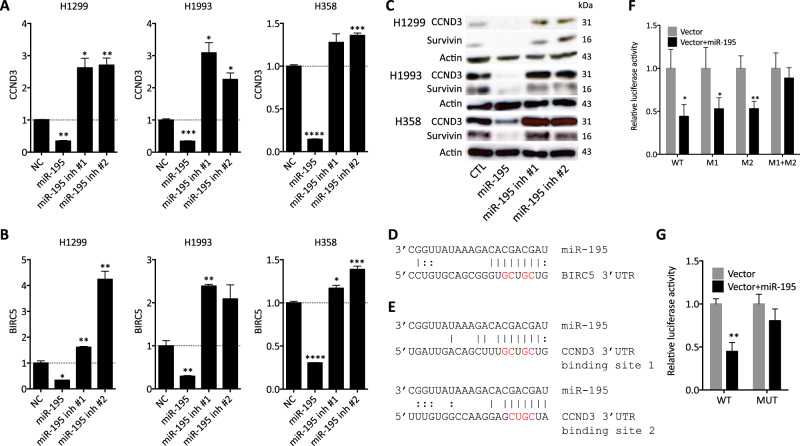
miR-195 directly targets *CCND3* and *BIRC5* in NSCLC cells. **a**,** b** mRNA levels of *CCND3* and *BIRC5* in NSCLC cells after transfection with miR-195 or inhibitors. **c** *CCND3* and *BIRC5* protein levels in NSCLC cells after transfection with miR-195 or inhibitors. **d**,** e** Predicted binding sites of miR-195 in the 3′UTRs of *BIRC5* and *CCND3*. Sites mutated or deleted as controls for the luciferase assay are shown in red. **f**, **g** Luciferase reporter assay for the direct and specific interaction of miR-195 with predicted target sites in the 3′UTRs of *CCND3* and *BIRC5*. Mutation of the target site in the *BIRC5* 3′UTR abolishes the inhibition of luciferase activity by miR-195. Only simultaneous mutation of two binding sites in the 3′UTR of *CCND3* abolishes the inhibition of luciferase activity by miR-195. WT wild-type, M1 mutation of *CCND3* 3′UTR binding site 1, M2 mutation of *CCND3* 3′UTR binding site 2, MUT mutation of *BIRC5* 3′UTR binding site. *p < 0.05; **p < 0.01; ***p < 0.001; ****p < 0.0001

We confirmed that mRNA and protein levels of *CCND3* and *BIRC5* are decreased by miR-195 and increased by miR-195 inhibitors in NSCLC cells (Fig. 3a–c). *BIRC5* is predicted to contain one miR-195 target site in its 3′UTR, while *CCND3* is predicted to contain two (Fig. 3d, e). We confirmed direct and specific regulation of *BIRC5* and *CCND3* by miR-195 using a luciferase reporter assay (Fig. 3f, g).

**Fig. 4:**
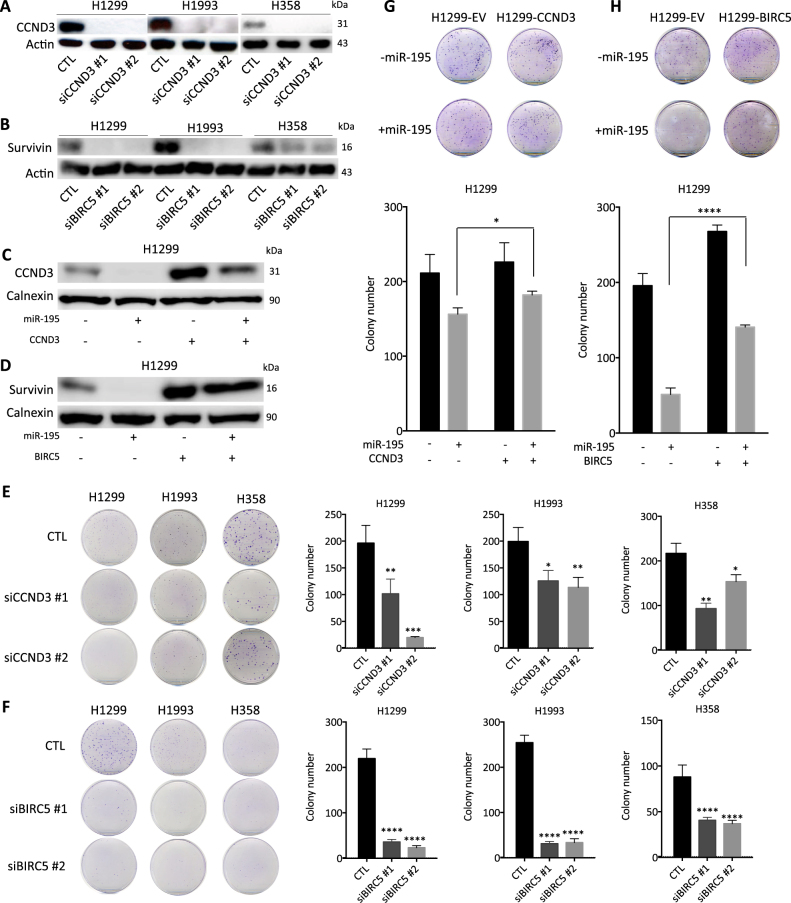
*CCND3* and *BIRC5* mediate the effect of miR-195 on NSCLC cell growth. **a−d** Protein levels of *CCND3* and *BIRC5*. **e**,** f** Colony formation of NSCLC cells after *CCND3* or *BIRC5* knockdown. **g** Rescue of miR-195-mediated inhibition of cell growth in *CCND3* overexpressing H1299 cells. **h** Rescue of miR-195-mediated inhibition of cell growth in *BIRC5* over-expressing H1299 cells. *p < 0.05; **p < 0.01; ***p < 0.001; ****p < 0.0001

To assess whether survivin and cyclin D3 mediate the function of miR-195 in NSCLC cells, we knocked down expression of each by siRNA. As predicted, knockdown of *CCND3* or *BIRC5* inhibits the growth of cancer cells (Fig. 4a, b, e, f). In contrast, overexpression of *CCND3* or *BIRC5* partially rescues the decrease in cell growth caused by miR-195 (Fig. 4c, d, g, h). Additionally, knockdown of *CCND3* causes cell cycle arrest at G1, and knockdown of *BIRC5* induces apoptosis and senescence (Supplementary Figure 6A, B; Fig. 2e, f, i). These observations support the roles of *CCND3* and *BIRC5* in mediating the regulation of NSCLC growth by miR-195.

Additionally, we demonstrated that miR-195 represses *CCND3* and *BIRC5* proteins in a pair of cell lines derived from the same patient, HBEC30-KT and HCC4017 (Supplementary Figure 7B). We did not observe induction of apoptosis by miR-195 in either HBEC30-KT or HCC4017 cells (Supplementary Figure 7C). We confirmed that miR-195 causes G1 phase arrest in both HBEC30-KT and HCC4017 cells (Supplementary Figure 7D), and that miR-195 induces senescence in HCC4017 cells, but not in HBEC30-KT cells (Supplementary Figure 7E).

### Correlation of *CCND3* and *BIRC5* with NSCLC patient survival

Analysis of gene expression in TCGA NSCLC patients shows that *CCND3* is expressed at slightly lower levels in tumor tissues compared to adjacent normal tissues in lung adenocarcinoma (LUAD) patients and lung squamous cell carcinoma (LUSC) patients (Supplementary Figure 8A). *CCND3* is positively correlated with miR-195 expression and its expression is not associated with overall survival or recurrence-free survival of LUAD and LUSC patients (Supplementary Figure 8B−D). This suggests that miR-195 may not be a major regulator of *CCND3* and that *CCND3* is mainly regulated by other genes in the context of NSCLC, including those regulated by, and others independent of, miR-195. It is also possible that both miR-195 and *CCND3* are regulated by the same group of genes in NSCLC.

**Fig. 5:**
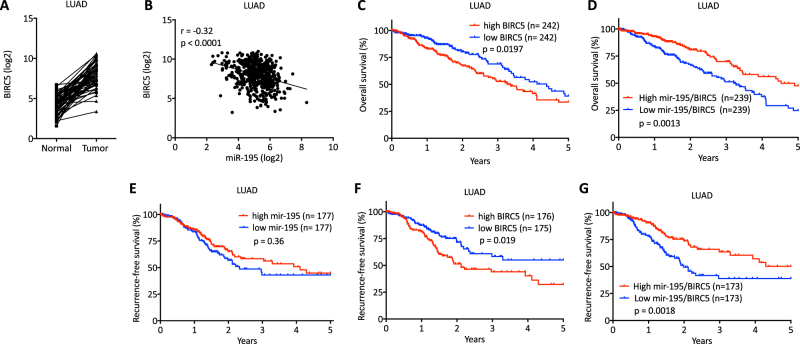
Correlation of *BIRC5* with miR-195 expression and patient survival in LUAD. **a** Expression of *BIRC5* is higher in tumor tissues than in adjacent normal tissues in 55 out of 57 LUAD patients. **b** Correlation of miR-195 expression with *BIRC5* expression in tumor tissues of LUAD patients (*n* = 467). **c** Overall survival curves for LUAD patients based on *BIRC5* expression in tumor tissues. **d** Overall survival curves for LUAD patients based on the ratio of miR-195 to *BIRC5* expression in tumor tissues. **e** Recurrence-free survival curves for LUAD patients based on miR-195 expression in tumor tissues. **f** Recurrence-free survival curves for LUAD patients based on *BIRC5* expression in tumor tissues. **g** Recurrence-free survival curves for LUAD patients based on the ratio of miR-195 to *BIRC5* expression in tumor tissues

*BIRC5*, however, is expressed at higher levels in tumor tissues compared to adjacent normal tissues in 98% (56 out of 57) of LUAD and 98% (50 out of 51) of LUSC patients (Fig. 5a, Supplementary Figure 8E). *BIRC5* levels are negatively correlated with miR-195 levels in both LUAD and LUSC patients and high expression of *BIRC5* is associated with worse overall survival and recurrence-free survival in LUAD patients but not in LUSC patients (Fig. 5b, c, f**;** Supplementary Figure 8F, G). These analyses indicate the importance of the miR-195/BIRC5 axis in NSCLC, especially in LUAD. To better evaluate this relationship, we stratified NSCLC patients from TCGA into two groups based on the ratio of miR-195 expression to *BIRC5* expression in tumor tissues. A high ratio of miR-195 to *BIRC5* in tumor tissues is significantly correlated with better overall survival and recurrence-free survival in LUAD, but not LUSC (Fig. 5d, g**;** Supplementary Figure 8H).

## Discussion

The dysregulation and tumor suppressor role of miR-195 has been demonstrated in various cancers. Recently, miR-195 has been reported to be downregulated in NSCLC and to repress the growth and progression of NSCLC cells^8, 13, 26, 27^. However, many questions regarding the function of miR-195 in NSCLC have not been answered. First, it has not been demonstrated whether loss of miR-195 is oncogenic in NSCLC. Second, regulation of cell cycle progression is the only demonstrated mechanism by which miR-195 represses NSCLC cell growth. It is not clear whether miR-195 regulates other pathways in NSCLC cells, such as apoptosis and senescence. Third, *MYB*, *CHEK1*, *HDGF*, and *IGF1R* are the only reported targets of miR-195 in NSCLC^8, 13, 26, 27^. It is not clear whether these targets are the most important in NSCLC, or whether other targets mediate the function of miR-195 in NSCLC as well.

In this study, we combine an HTS and statistical analysis of TCGA data to re-identify miR-195 as a tumor suppressor in NSCLC. We demonstrate that repression of miR-195 accelerates NSCLC growth, while activation of miR-195 represses NSCLC growth *in vitro* and* in vivo*. We also show that miR-195 causes G1-phase cell cycle arrest, apoptosis, and senescence in NSCLC cells. Additionally, we identify two targets (*CCND3* and *BIRC5*) of miR-195 in NSCLC cells.

Dysregulation of cell cycle progression is a common feature of all types of cancer^35^. Cyclin D3 is an important regulator of G1/S cell cycle progression; inhibition of *CCND3* causes G1 phase arrest^36^. miR-195 has been shown to regulate cancer cell growth by targeting *CCND3* in hepatocellular carcinoma^17^ and glioblastoma^14^. Here, we report that miR-195 directly targets *CCND3* to regulate the growth of NSCLC cells, highlighting the potential of miR-195 to target the dysregulated cell cycle of NSCLC cells.

*BIRC5*, a well-characterized oncogene that codes for survivin, has been found to be aberrantly overexpressed in a variety of cancers^37, 38^. Survivin regulates the growth, survival, migration, and invasion of cancer cells and even plays a role in cellular senescence^32, 33, 39, 40^. Targeting survivin has been proposed to be an attractive strategy for cancer treatment^41, 42^. Survivin has been shown to be downregulated by miR-195 in endometrial stromal cells but was not validated as a direct target of miR-195^43^. *BIRC5* was first shown to be a direct target of miR-195 in bladder cancer^44^. Here, we demonstrate that miR-195 directly targets *BIRC5* to regulate the apoptosis and senescence of NSCLC cells. These results collectively indicate that the miR-195/survivin axis is one of the major regulators of NSCLC growth, and therefore a candidate target for NSCLC treatment. Importantly, miR-195 and *BIRC5* expression are significantly associated with lung adenocarcinoma patient survival, but not squamous cell carcinoma patient survival, suggesting that the miR-195/survivin axis might be a more important prognostic marker and therapeutic target in lung adenocarcinoma. We also found that the ratio of miR-195 to *BIRC5* in patient tumors is associated with overall survival and recurrence-free survival of lung adenocarcinoma patients, but not lung squamous cell carcinoma patients. Given that most NSCLC, including adenocarcinoma and squamous cell carcinoma, are treated similarly even though the tumors are histologically different, the relevance of the miR-195/survivin axis in lung adenocarcinoma could provide a new mechanism for differentiating lung adenocarcinoma from squamous cell carcinoma and for developing treatments specific for the former.

In conclusion, we demonstrated that miR-195 inhibits the growth of NSCLC cells by regulating cell cycle progression, apoptosis, and senescence, that miR-195 suppresses NSCLC, at least partially, through targeting *CCND3* and *BIRC5*, and that dysregulation of the miR-195/BIRC5 axis may contribute to the progression of lung adenocarcinoma, establishing the relevance of levels of miR-195 and *BIRC5* as prognostic factors and of the miR-195/BIRC5 axis as a therapeutic target for lung adenocarcinoma.

## Materials and methods

### Reagents

miR-195 mimics were purchased from GE Dharmacon (Colorado) and IDT (Iowa). Two negative control oligos were purchased from Dharmacon: D-001810-10-05 (CTL) and CN-001000-01-20 (NC). Two miR-195 inhibitors, IH-300643-05-0005 (miR-195 inh #1) and HSTUD0320 (miR-195 inh #2), were obtained from Dharmacon and Sigma-Aldrich (Missouri). Lipofectamine RNAiMAX and 2000 were purchased from Thermo Fisher (Massachusetts). Oligos were transfected into cells at 25 nm unless otherwise specified.

Two siRNAs for *CCND3* were purchased from Sigma-Aldrich:

SASI_Hs01_00050184 targeting TGGTCAAAAAGCATGCCCAGA, and

SASI_Hs01_00050186 targeting CCTAGGGAAGCTCAAGTGGGA.

Two siRNAs for *BIRC5* were purchased from Sigma-Aldrich:

SASI_Hs01_00052228 targeting ACTTGGCCCAGTGTTTCTTCT, and

SASI_Hs01_00052229 targeting GTGTCTGGACCTCATGTTGTT.

All NSCLC cell lines were established at the NCI. HBECs were established at the Hamon Center for Therapeutic Oncology Research at UT Southwestern Medical Center in Dallas, Texas. IMR-90 cells were purchased from ATCC. WI-38 cells were a gift from Dr. P. Renee Yew. All cancer cell lines were grown in RPMI-1640 medium supplemented with 5% fetal bovine serum (FBS). HBECs were grown in keratinocyte serum-free medium (KSFM) supplemented with bovine pituitary extract and human recombinant epidermal growth factor. IMR-90 and WI-38 were grown in Dulbecco's modified Eagle's medium (DMEM) supplemented with 10% FBS and 1% penicillin/streptomycin. All cell lines were authenticated using short tandem repeat profiling, and confirmed to be mycoplasma-free through PCR.

Luciferase-pcDNA3 plasmid was a gift from William Kaelin (Addgene plasmid # 18964). Mir-X™ Inducible miRNA System was purchased from Clontech (California). pLenti-III-miR-Off-control and LentimiRa-Off-hsa-miR-195-5P were obtained from ABM (Canada). pSpCas9(BB)-2A-GFP (PX458) (Addgene plasmid # 48138) and pSpCas9(BB)-2A-Puro (PX459) V2.0 (Addgene plasmid # 62988) were gifts from Feng Zhang. Two sgRNAs were cloned into PX458 and PX459 separately and co-transfected into H1299 cells.

Sequences for sgRNA1 were:

sgRNA1-forward: CACCGGGTGGTGAAAACTACCGAGG and

sgRNA1-reverse: AAACCCTCGGTAGTTTTCACCACCC.

Sequences for sgRNA2 were:

sgRNA2-forward: CACCGTTGAGGCAGAACTTACTCCC and

sgRNA2-reverse: AAACGGGAGTAAGTTCTGCCTCAAC.

### High-throughput screen

An miRNA library was obtained from GE Dharmacon (CS-001010 Human Mimics Lot 09167 and CS-001015 Supplement Human Mimic 16.0 Lot 11144). The library was arrayed in one-mimic/one-well format in the central 60 wells of 96-well plates. Reverse transfections of mimics into NSCLC cells (H358, H1993, and H1155) were performed in triplicate. After incubation for a total of 120 h, cell viability was assayed using the CellTiter-Glo^®^ Assay (Promega, Wisconsin). Each miRNA mimic was assigned a relative viability calculated by normalizing replicate means to the mean of the central 60 wells on each plate.

### Colony formation assay

500−1000 cells were seeded on 10 cm dishes. Medium was replaced every 3−4 days. After 10−30 days (depending on the cell type), colonies were visualized by staining with 1% crystal violet and washing with tap water. Pictures were taken and the number of colonies was counted using ImageJ (NIH).

### RNA extraction and qRT-PCR

Total RNA was prepared using the mirVana miRNA Isolation Kit (Ambion). RNU44 was used as a control for miRNA normalization. Glyceraldehyde 3-phosphate dehydrogenase (GAPDH) expression was used as a control for mRNA normalization.

Primers used for GAPDH:

forward-GAAGGTGAAGGTCGGAGTC and

reverse-GAAGATGGTGATGGGATTTC.

Primers for *CCND3*:

forward-CTTACTGGATGCTGGAGGTATG and

reverse-CGGGTACATGGCAAAGGTATAA.

Primers for *BIRC5*:

forward-GCACCACTTCCAGGGTTTAT and

reverse-CAGACGCTTCCTATCACTCTATTC.

### miRNA expression profiling

H358 and H1993 cells were transfected with miR-195 mimic or mock transfected. Twenty-four hours later, total RNA was prepared as described above. Gene expression profiling was assessed by microarray using the HumanHT-12v4 Expression BeadChip (Illumina).

### Luciferase reporter assay

The segments of the wild-type 3′UTRs of genes of interest containing predicted target sites of miR-195 were cloned into the pmirGLO dual-luciferase reporter (Promega). Mutant constructs were generated with the seed target sites mutated or deleted as specified above under the “Results” section. Firefly luciferase was used as the primary reporter for miRNA regulation of the 3′UTR. Renilla luciferase was used as an internal control for normalization. H1299 cells were maintained in 96-well plates and co-transfected with luciferase reporters (0.05 μg/well) and miRNA mimics or control oligo (15 nm). Luciferase activities were measured after 72 h using the Dual-Glo Luciferase Assay System (Promega). Firefly luciferase activity was normalized to sea pansy luciferase activity to evaluate the regulatory effect of miR-195 on its putative targets.

Primers for *CCND3* 3′UTR:

forward-CGAGCTCGCTAGCCTCGAGGCTCCTCTCAGTACTTTGGAGGC and

reverse-GGTCGACTCTAGACTCGACCTGTGTCAACAGGGCTTGCCT.

Primers for *CCND3* 3′UTR with mutations in the first binding site of miR-195:

forward-TGTGATTGACAGCTTTAATCGTGAAGGCTCATTTTAATTTATTAATTGC and

reverse-TGAGCCTTCACGATTAAAGCTGTCAATCACACAGGAGAA.

Primers for *CCND3* 3′UTR with mutations in the second binding site of miR-195:

forward-GCCAAGGAGAACGTATAGCCTGGGGTGGGGTCATG and

reverse-AGGCTATACGTTCTCCTTGGCCACAAAGATCCTTTTG.

Primers for *BIRC5* 3′UTR:

forward-TAGCTGAGAGCTCGGCCTCTGGCCGGAGC and

reverse-GGTCTGATCTAGAGGAAGGCTCTGCCCACGCG.

Primers for *BIRC5* 3′UTR with mutations in the binding site of miR-195:

forward-CTGCCTGTGCAGCGGGTCGTCGTGGTAACAGTGGCTGCTTCTCTCTCTC and

reverse-GAGAGAGAGAAGCAGCCACTGTTACCACGACGACCCGCTGCACAGGCAG.

### Cell cycle analysis

After the indicated treatments, cells were detached and collected by centrifuging at 1000 rpm for 5 min. The cells were washed once with phosphate-buffered saline (PBS) and fixed with 70% ethanol at −20 °C. Cells were harvested by centrifugation at 1500 rpm for 5 min at 4 °C. Cells were then re-suspended in 0.5% Triton-X100 containing 50 μg/mL propidium iodide and 100 μg/mL RNase A and incubated for 40 min at 37 °C. Cell cycle data were collected on a Cytomics FC 500 flow cytometer (Beckman Coulter), with 5000−20,000 events collected per sample. Data were analyzed using FlowJo v10 (TreeStar).

### Cell apoptosis and growth rate assays

Cells were plated in 96-well plates and treated as specified. After 18−24 h, CellPlayer Caspase 3/7 Reagent (Essen BioScience) was added and apoptotic events were captured using an IncuCyte FLR imaging system (Essen). Cell confluence was monitored at the same time. After 4−5 days, the total number of cells in each well was determined by staining for total DNA content using Vybrant DyeCycle Green DNA stain (Invitrogen). The percentage of apoptotic cells was determined from the ratio of apoptotic events to the total number of cells, with the latter estimated by combining the DNA content assay at the end point and the cell confluence at each time point. Cell growth curves were derived from the observed confluence at each time point.

### Senescence-associated β-galactosidase staining

Cells were transfected with oligos (25 nm) and cultured for 4−5 days. Cells were then washed with PBS three times and fixed with 4% paraformaldehyde for 10 min at room temperature. Cells were then washed with PBS (PH 6.0) three times and stained with staining agents (1.2 mm potassium ferricyanide + 1.2 mm potassium ferrocyanide + 1 mm X-Gal) overnight at 37 °C. Pictures were taken from five randomly selected visual fields and senescent cells (stained blue) were counted using ImageJ.

### Western blots

Cell lysates were prepared using radioimmunoprecipitation assay (RIPA) buffer. Equal amounts of lysate were resolved by sodium dodecyl sulfate polyacrylamide gel electrophoresis (SDS-PAGE) and transferred to ImmunBlot polyvinylidine difluoride (PVDF) membranes (Bio-Rad). Membranes were blocked and probed with specific primary antibodies. Antibodies to p53 (#2527S), p21 (#2947S), actin (#4970P), survivin (#2808T), phospho-Rb (#8180), vimentin (#5471), parp (#9542P) were purchased from Cell Signaling Technology (MA, USA). Antibodies to calnexin (sc-11397), ccnd3 (sc-182) were purchased from Santa Cruz Biotechnology (TX, USA). Antibody to Rb (#554136) was purchased from BD Pharmingen (CA, USA). E-cadherin (#131900) antibody was purchased from Thermo Fisher Scientific (TX, USA). Bound antibodies were detected with secondary antibodies conjugated with horseradish peroxidase (Santa Cruz Biotechnology) and visualized by enhanced chemiluminescent substrate (Pierce/Thermo Fisher) on an Odyssey Fc Imaging system (LI-COR).

### Animal experiments

Six- to seven-week-old female athymic nude Foxn1nu (nu/nu) mice were subcutaneously injected with 1×10^6^ cells in 200 μL PBS/matrigel (v/v, 1:1). Tumor volumes were measured using calipers and calculated by the formula: volume = (width)^2^ × length/2. When tumor reached about 50−100 mm^3^, mice were treated as specified. Animal protocols were approved by the Institutional Animal Care and Use Committee of the University of Texas Health Science Center at San Antonio.

### Statistical analysis

Statistical analyses, including Student’s *t*-test and two-way ANOVA, were performed using GraphPad Prism, with *p* < 0.05 considered statistically significant.

## Electronic supplementary material

Supplementary Materials and Methods

Supplementary Figure Legends

Supplementary Figure 1

Supplementary Figure 2

Supplementary Figure 3

Supplementary Figure 4

Supplementary Figure 5

Supplementary Figure 6

Supplementary Figure 7

Supplementary Figure 8

Supplementary Table 1

Supplementary Table 2

Supplementary Table 3

Supplementary Table 4

Supplementary Table 5
