# Supplementary material for: miR-195 targets cyclin D3 and survivin to modulate the tumorigenesis of non-small cell lung cancer
Source: Cell Death Dis. 2018 Feb 7;9(2):193. doi: 10.1038/s41419-017-0219-9 (PMC5833354; doi:10.1038/s41419-017-0219-9)
Supplement: Supplementary file 1 — Supplementary Materials and Methods [file 41419_2017_219_MOESM1_ESM.docx]

**SUPPLEMENTARY MATERIALS AND METHODS**

**Cell viability assay.** Cells were plated in 96-well format and transfected with oligos in different concentrations. After incubating for 96-120 h, when cells were close to confluence, cell viability was determined using the CellTiter-Glo® Assay. Dose-response curves were generated using GraphPad Prism 7 (California).

**RNA extraction and qRT-PCR.** Total RNA was prepared using the mirVana miRNA Isolation Kit (Ambion) according to manufacturer instructions. Intracellular miRNA levels were assessed by qRT-PCR using miRNA expression assay primer and probe sets (Applied Biosystems). RNU44 was used as a control for miRNA normalization. Threshold cycle times (Ct) were obtained and relative gene expression was calculated using the comparative cycle time method.

**Western blots.** Cell lysates were prepared using RIPA buffer. For electrophoresis, equal amounts of cell lysate were resolved by SDS-PAGE and transferred to ImmunBlot PVDF membranes (Bio-Rad). Membranes were blocked and probed with specific primary antibodies. Bound antibodies were detected with secondary antibodies conjugated with horseradish peroxidase (HRP) (Santa Cruz Biotechnology) and visualized by enhanced chemiluminescent (ECL) substrate (Pierce/Thermo Fisher) on an Odyssey Fc Imaging System (LI-COR).

**Measurement of oxygen consumption rate (OCR) and extracellular acidification rate (ECAR).** OCR and ECAR measurements were performed using the XF96 Extracellular Flux analyzer (Seahorse Bioscience, North Billerica, MA) per manufacturer instructions.
