# Supplementary material for: miR-195 targets cyclin D3 and survivin to modulate the tumorigenesis of non-small cell lung cancer
Source: Cell Death Dis. 2018 Feb 7;9(2):193. doi: 10.1038/s41419-017-0219-9 (PMC5833354; doi:10.1038/s41419-017-0219-9)
Supplement: Supplementary file 2 — Supplementary Figure Legends [file 41419_2017_219_MOESM2_ESM.docx]

**SUPPLEMENTARY FIGURE LEGENDS**

**Supplementary Figure 1. miR-195 is a tumor suppressor in NSCLC. (A)** The expression of miR-195 in tumor tissues compared to adjacent normal tissues in lung adenocarcinoma (LUAD) and squamous cell carcinoma (LUSC) patients. **(B,C)** Overall survival curves for LUAD and LUSC patients. Patients with miR-195 levels in the top 50% and bottom 50% were selected from 386 LUAD and 302 LUSC patients, defining high and low mir-195 expression groups **(D)** Relative miR-195 expression in normal cell lines and cancer cell lines.

**Supplementary Figure 2. miR-195 represses NSCLC cell growth. (A)** Dose-response curves for transfection with miR-195 mimic in different cell lines. **(B)** Levels of miR-195 in H1299, H1993 and H358 cells after transfection with miR-195 mimic or inhibitors. **(C)** Colony formation results in H1299, H1993 and H358 cells transfected with miR-195 mimic or inhibitors. *, p<0.05; **, p<0.01; ***, p<0.001; ****, p<0.0001.

**Supplementary Figure 3. Expression of fluorescent reporters. (A-B)** Doxycycline induces expression of ZsGreen. **(C-D)** Nearly 100% of H1299 cells were infected with lentivirus over-expressing miR-195 inhibitor or control lentivirus, as indicated by GFP fluorescence.

**Supplementary Figure 4. (A)** Representative bioluminescence images of xenografts bearing H1299/luc-EV cells or H1299/luc-miR-195 cells. **(B)** Representative images of tumors generated from H1299/ptet-miR-195 cells treated with or without doxycycline (dox).

**Supplementary Figure 5. miR-195 has no effect on mitochondrial respiration. (A,B)** Oxygen consumption rate (OCR) and extracellular acidification rate (ECAR) in A549 cells under different conditions were measured on a Seahorse XFe96 Analyzer.

**Supplementary Figure 6. CCND3 and BIRC5 mediate the effects of miR-195 in NSCLC cells. (A)** Cell cycle distribution as a function of *CCND3* knock-down in NSCLC cells. **(B)** Induction of apoptosis in NSCLC cells after *BIRC5* knock-down.

**Supplementary Figure 7. miR-195 regulates cell cycle and senescence in normal and cancer cells. (A)** Relative miR-195 expression in HBEC30-KT and HCC3017 cells. **(B)** miR-195 represses protein levels of CCND3 and survivin in HBEC30-KT and HCC4017 cells. **(C)** miR-195 has no effect on apoptosis in HBEC30-KT and HCC4017 cells. **(D)** Cell cycle distribution as a function of miR-195 in HBEC30-KT and HCC4017 cells. **(E)** β-gal staining images and quantification of cells transfected with miR-195 mimic. ***, p<0.001.

**Supplementary Figure 8. Correlation of *CCND3* and *BIRC5* with miR-195 expression and patient survival. (A)** Expression of *CCND3* is slightly lower in tumor tissues than in adjacent normal tissues in both LUAD and LUSC patients. **(B)** Correlation of miR-195 expression with *CCND3* expression in tumor tissues of LUAD and LUSC patients. **(C)** Overall survival curves for LUAD and LUSC patients based on *CCND3* expression in tumor tissues. **(D)** Recurrence-free survival curves for LUAD and LUSC patients based on *CCND3* expression in tumor tissues. **(E)** Expression of *BIRC5* is higher in tumor tissues than in adjacent normal tissues in 50 out of 51 LUSC patients. **(F)** Correlation of miR-195 expression with *BIRC5* expression in tumor tissues of LUSC patients. **(G)** Overall survival and recurrence-free survival curves for LUSC patients based on *BIRC5* expression in tumor tissues. **(H)** Overall survival and recurrence-free survival curves for LUSC patients based on the ratio of miR-195 to *BIRC5* in tumors. **(I)** Recurrence-free survival curves for LUSC patients based on miR-195 expression in tumor tissues. ****, p < 0.0001.
