# Supplementary figures and images for: miR-195 targets cyclin D3 and survivin to modulate the tumorigenesis of non-small cell lung cancer
Source: Cell Death Dis. 2018 Feb 7;9(2):193. doi: 10.1038/s41419-017-0219-9 (PMC5833354; doi:10.1038/s41419-017-0219-9)

**Supplementary Figure 1**

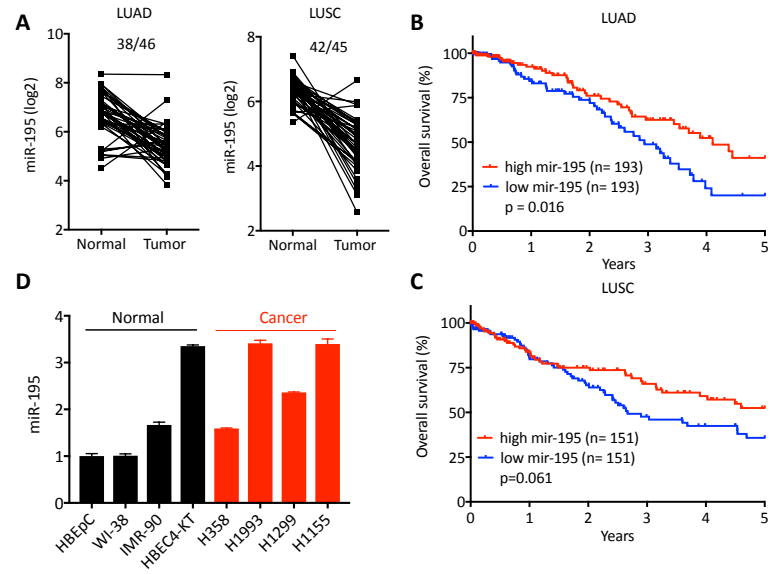

Supplement: Supplementary file 3 — Supplementary Figure 1 [file 41419_2017_219_MOESM3_ESM.pdf]

**Supplementary Figure 2**

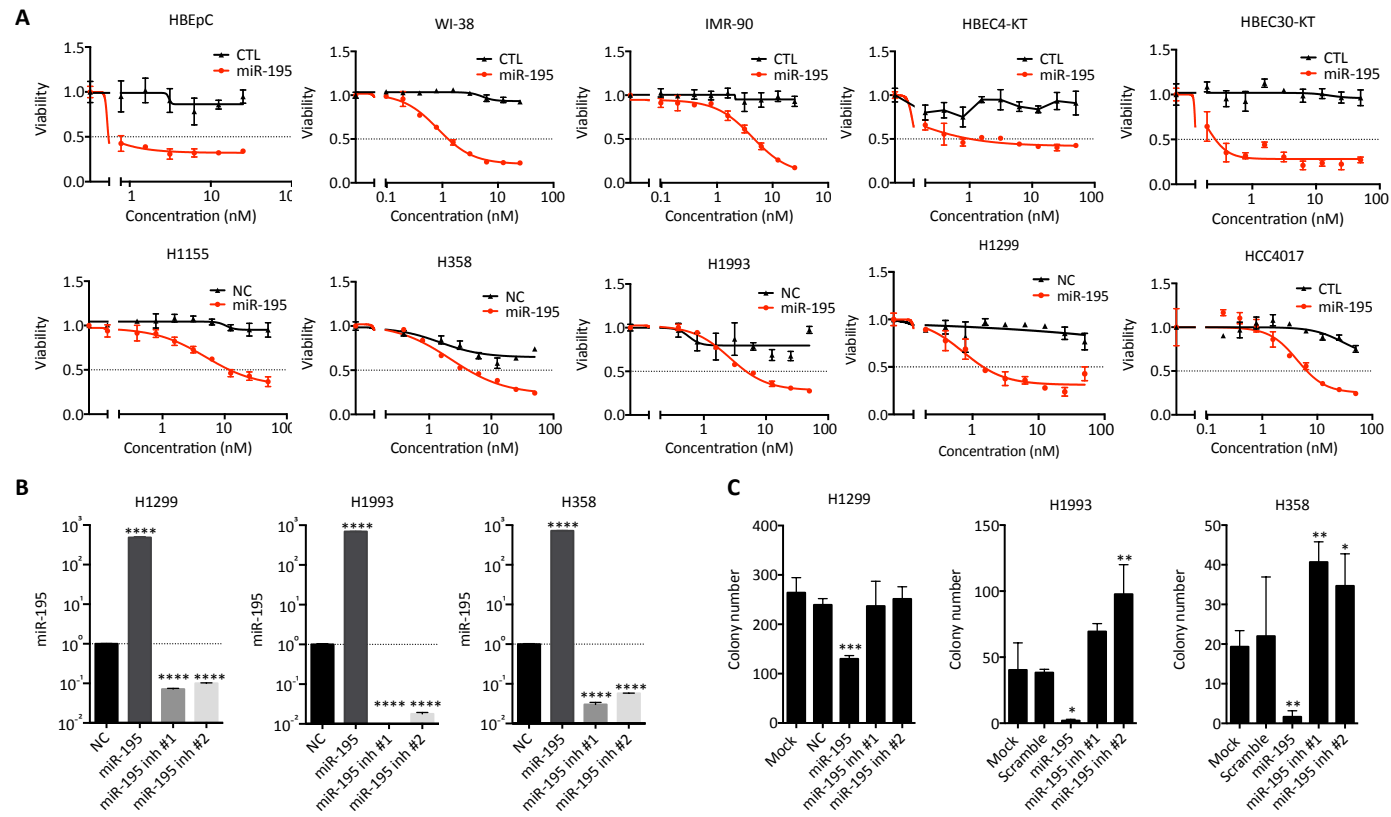

Supplement: Supplementary file 4 — Supplementary Figure 2 [file 41419_2017_219_MOESM4_ESM.pdf]

Supplementary Figure 3

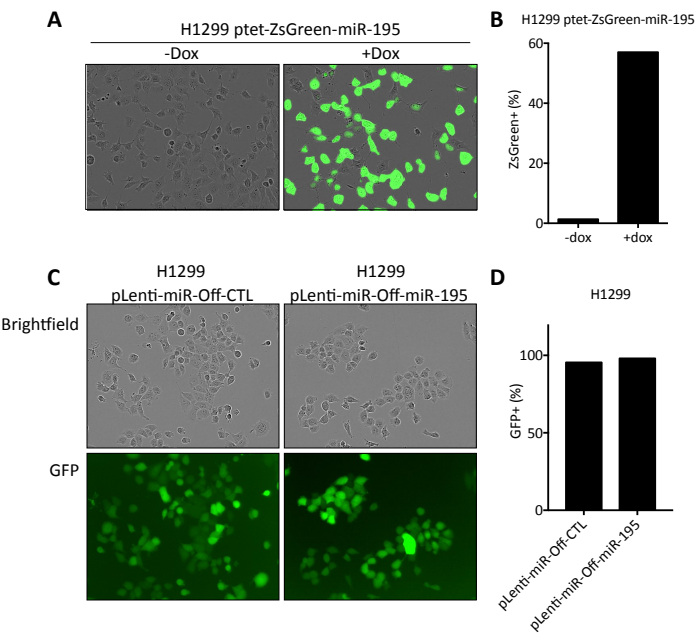

Supplement: Supplementary file 5 — Supplementary Figure 3 [file 41419_2017_219_MOESM5_ESM.pdf]

**Supplementary Figure 4**

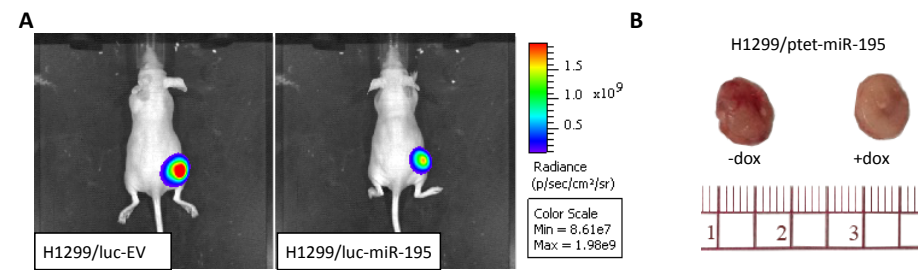

Supplement: Supplementary file 6 — Supplementary Figure 4 [file 41419_2017_219_MOESM6_ESM.pdf]

Supplementary Figure 5

A

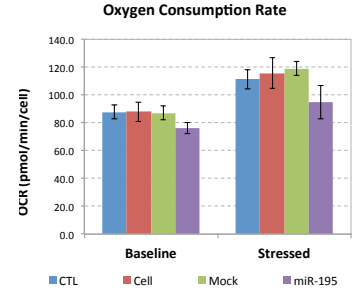

B

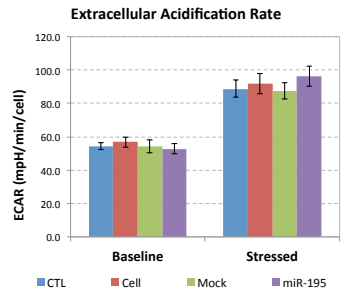

Supplement: Supplementary file 7 — Supplementary Figure 5 [file 41419_2017_219_MOESM7_ESM.pdf]

### Supplementary Figure 6

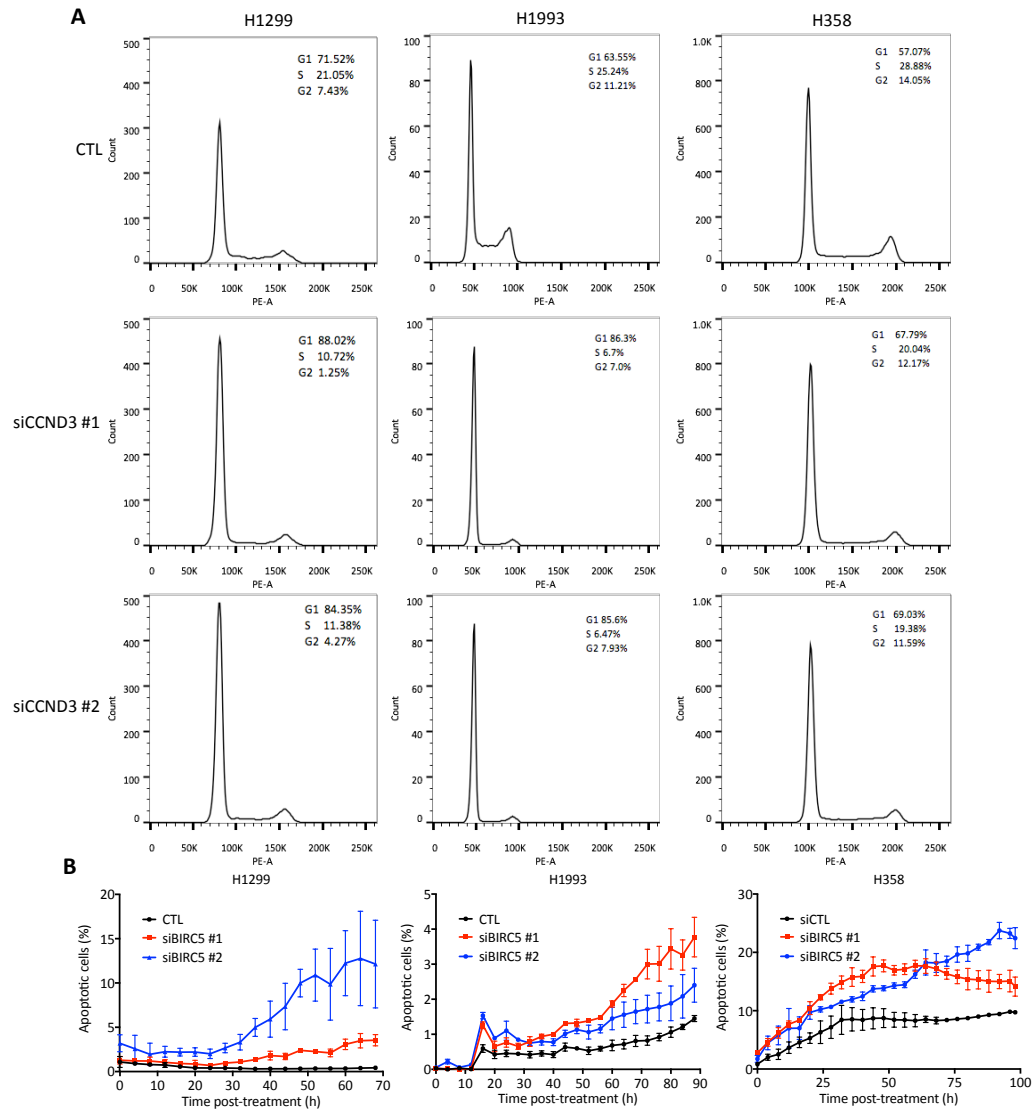

Supplement: Supplementary file 8 — Supplementary Figure 6 [file 41419_2017_219_MOESM8_ESM.pdf]

Supplementary Figure 7

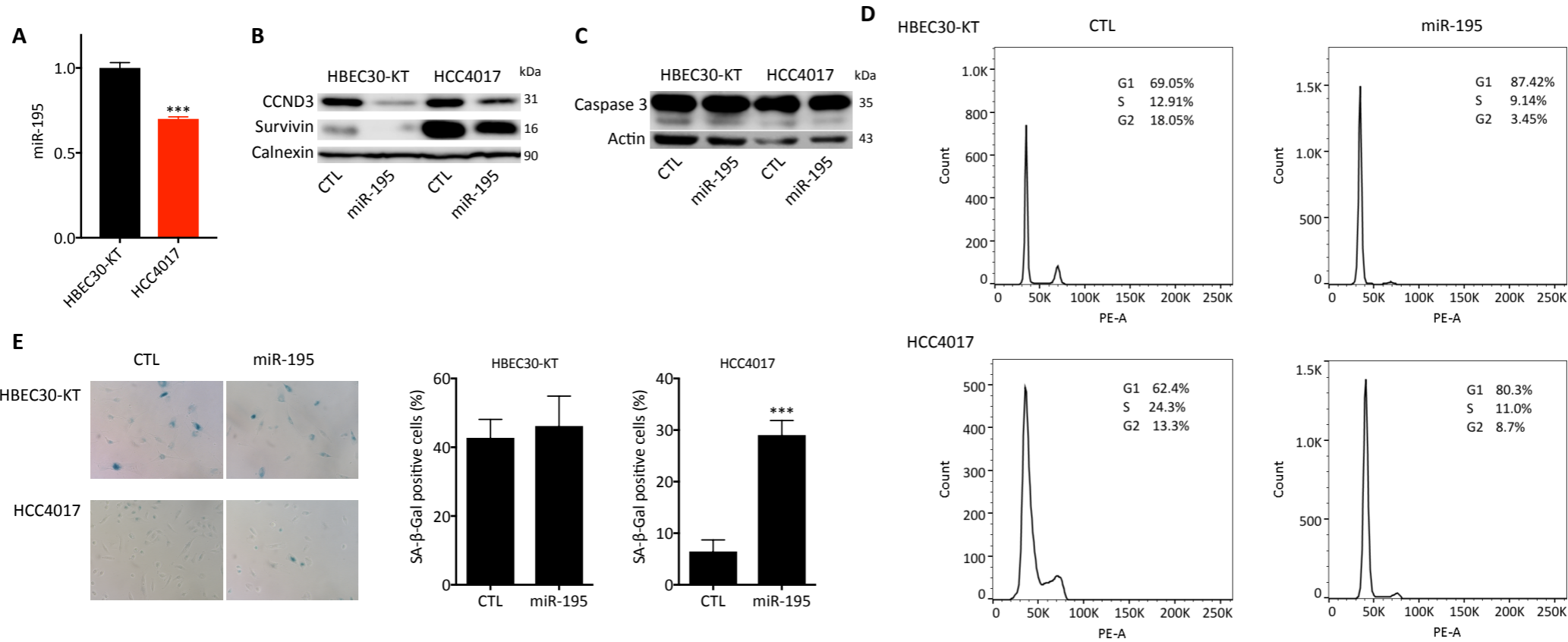

Supplement: Supplementary file 9 — Supplementary Figure 7 [file 41419_2017_219_MOESM9_ESM.pdf]

**Supplementary Figure 8**

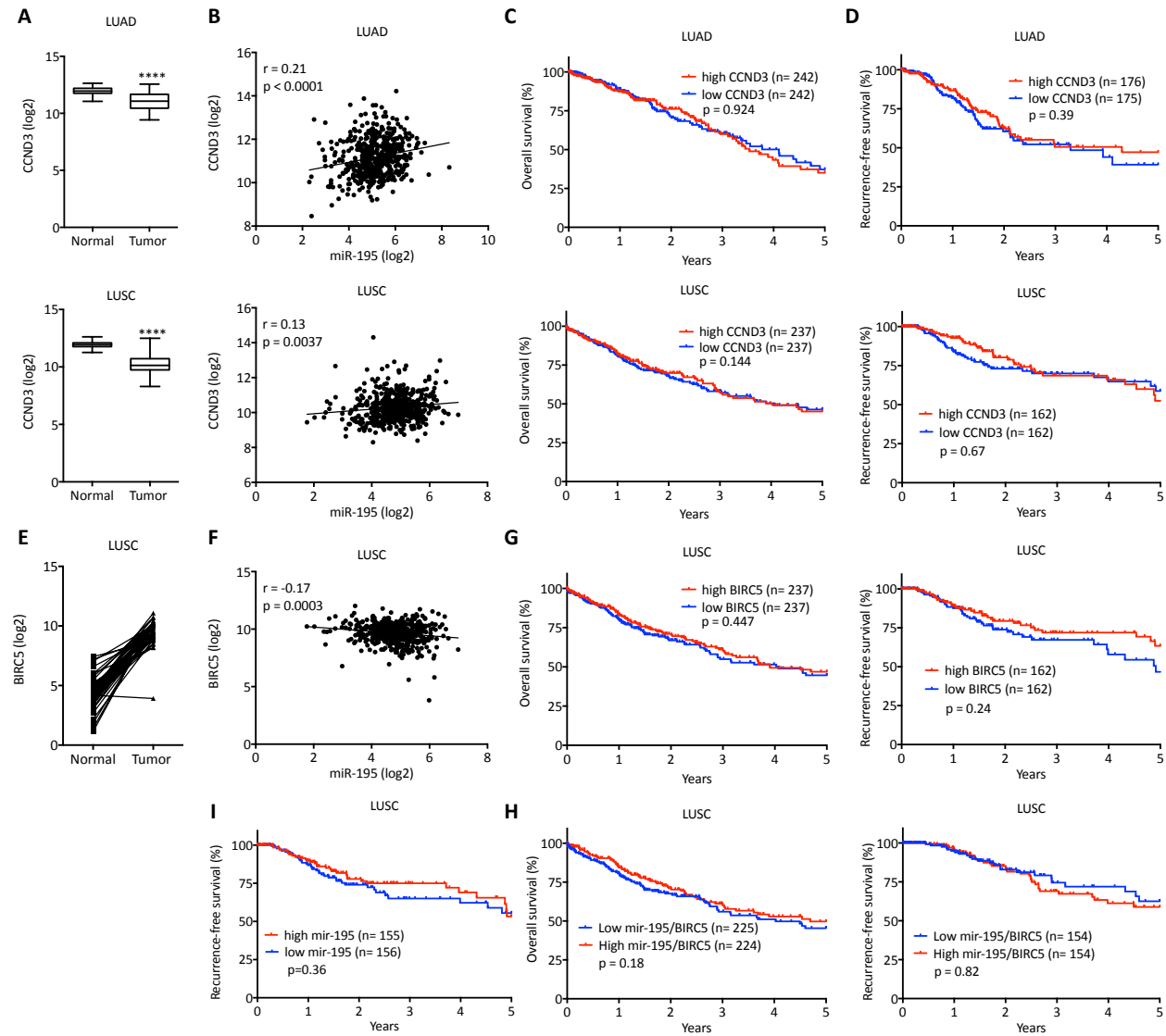

Supplement: Supplementary file 10 — Supplementary Figure 8 [file 41419_2017_219_MOESM10_ESM.pdf]
