# Supplementary material for: miR-195 targets cyclin D3 and survivin to modulate the tumorigenesis of non-small cell lung cancer
Source: Cell Death Dis. 2018 Feb 7;9(2):193. doi: 10.1038/s41419-017-0219-9 (PMC5833354; doi:10.1038/s41419-017-0219-9)
Supplement: Supplementary file 13 — Supplementary Table 3 [file 41419_2017_219_MOESM13_ESM.docx]

**Supplementary Table 3. Reported targets of miR-195 in cancer.**

| **Target** | **Cancer type** | **References** | **Fold change in H358/H1993** |
| --- | --- | --- | --- |
| CCND3 | Glioblastoma, hepatocellular carcinoma (HCC) | 1, 2 | -2.54/-3.35 |
| CDC25A | HCC | 2 | -1.64/-2.47 |
| CDK4 | Bladder cancer, HCC | 2, 3 | -1.40/-1.98 |
| FASN | Osteosarcoma | 4 | -1.90/-1.46 |
| CCNE1 | Glioma, HCC | 2, 5, 6 | -1.57/-1.37 |
| CDC42 | Esophageal squamous cell carcinoma, HCC | 7, 8 | -1.71/-1.12 |
| CCND1 | Breast cancer, glioma, HCC, osteosarcoma | 6, 9, 10 | -1.34/-1.28 |
| WEE1 | Melanoma | 11 | -1.30/-1.30 |
| CDK6 | Gastric cancer, HCC | 10, 12 | -1.48/-1.06 |
| BCL2 | Colorectal cancer | 13 | -1.04/-1.31 |
| RAF1 | Breast cancer | 9 | 1.09/-1.23 |
| E2F3 | Glioblastoma, HCC | 1, 10 | 1.00/-1.02 |
| VEGF | HCC | 8 | -1.12/-1.26 |
| BTRC | HCC | 2 | -1.21/-1.52 |
| VAV2 | HCC | 8 | 1.11/ 1.08 |
| GLUT3 | Bladder cancer | 14 | N/A |
| CHEK1 | NSCLC | 15 | -2.27/-1.98 |
| MYB | NSCLC | 16 | -1.53/-1.45 |
| HDGF | NSCLC | 17 | -1.39/-1.22 |
| IGF1R | NSCLC | 18 | -1.13/ 1.02 |

1. Zhang QQ, Xu H, Huang MB, Ma LM, Huang QJ, Yao Q*, et al.* MicroRNA-195 plays a tumor-suppressor role in human glioblastoma cells by targeting signaling pathways involved in cellular proliferation and invasion. *Neuro Oncol* 2012, **14**(3)**:** 278-287.

2. Furuta M, Kozaki K, Tanimoto K, Tanaka S, Arii S, Shimamura T*, et al.* The tumor-suppressive miR-497-195 cluster targets multiple cell-cycle regulators in hepatocellular carcinoma. *PLoS One* 2013, **8**(3)**:** e60155.

3. Lin Y, Wu J, Chen H, Mao Y, Liu Y, Mao Q*, et al.* Cyclin-dependent kinase 4 is a novel target in micoRNA-195-mediated cell cycle arrest in bladder cancer cells. *FEBS Lett* 2012, **586**(4)**:** 442-447.

4. Mao JH, Zhou RP, Peng AF, Liu ZL, Huang SH, Long XH*, et al.* microRNA-195 suppresses osteosarcoma cell invasion and migration in vitro by targeting FASN. *Oncol Lett* 2012, **4**(5)**:** 1125-1129.

5. Sekiya Y, Ogawa T, Iizuka M, Yoshizato K, Ikeda K, Kawada N. Down-regulation of cyclin E1 expression by microRNA-195 accounts for interferon-beta-induced inhibition of hepatic stellate cell proliferation. *J Cell Physiol* 2011, **226**(10)**:** 2535-2542.

6. Hui W, Yuntao L, Lun L, WenSheng L, ChaoFeng L, HaiYong H*, et al.* MicroRNA-195 inhibits the proliferation of human glioma cells by directly targeting cyclin D1 and cyclin E1. *PLoS One* 2013, **8**(1)**:** e54932.

7. Fu MG, Li S, Yu TT, Qian LJ, Cao RS, Zhu H*, et al.* Differential expression of miR-195 in esophageal squamous cell carcinoma and miR-195 expression inhibits tumor cell proliferation and invasion by targeting of Cdc42. *FEBS Lett* 2013, **587**(21)**:** 3471-3479.

8. Wang R, Zhao N, Li S, Fang JH, Chen MX, Yang J*, et al.* MicroRNA-195 suppresses angiogenesis and metastasis of hepatocellular carcinoma by inhibiting the expression of VEGF, VAV2, and CDC42. *Hepatology* 2013, **58**(2)**:** 642-653.

9. Li D, Zhao Y, Liu C, Chen X, Qi Y, Jiang Y*, et al.* Analysis of MiR-195 and MiR-497 expression, regulation and role in breast cancer. *Clin Cancer Res* 2011, **17**(7)**:** 1722-1730.

10. Xu T, Zhu Y, Xiong Y, Ge YY, Yun JP, Zhuang SM. MicroRNA-195 suppresses tumorigenicity and regulates G1/S transition of human hepatocellular carcinoma cells. *Hepatology* 2009, **50**(1)**:** 113-121.

11. Bhattacharya A, Schmitz U, Wolkenhauer O, Schonherr M, Raatz Y, Kunz M. Regulation of cell cycle checkpoint kinase WEE1 by miR-195 in malignant melanoma. *Oncogene* 2013, **32**(26)**:** 3175-3183.

12. Deng H, Guo Y, Song H, Xiao B, Sun W, Liu Z*, et al.* MicroRNA-195 and microRNA-378 mediate tumor growth suppression by epigenetical regulation in gastric cancer. *Gene* 2013, **518**(2)**:** 351-359.

13. Liu L, Chen L, Xu Y, Li R, Du X. microRNA-195 promotes apoptosis and suppresses tumorigenicity of human colorectal cancer cells. *Biochem Biophys Res Commun* 2010, **400**(2)**:** 236-240.

14. Fei X, Qi M, Wu B, Song Y, Wang Y, Li T. MicroRNA-195-5p suppresses glucose uptake and proliferation of human bladder cancer T24 cells by regulating GLUT3 expression. *FEBS Lett* 2012, **586**(4)**:** 392-397.

15. Liu B, Qu J, Xu F, Guo Y, Wang Y, Yu H*, et al.* MiR-195 suppresses non-small cell lung cancer by targeting CHEK1. *Oncotarget* 2015, **6**(11)**:** 9445-9456.

16. Yongchun Z, Linwei T, Xicai W, Lianhua Y, Guangqiang Z, Ming Y*, et al.* MicroRNA-195 inhibits non-small cell lung cancer cell proliferation, migration and invasion by targeting MYB. *Cancer Lett* 2014, **347**(1)**:** 65-74.

17. Guo H, Li W, Zheng T, Liu Z. MiR-195 targets HDGF to inhibit proliferation and invasion of NSCLC cells. *Tumour Biol* 2014, **35**(9)**:** 8861-8866.

18. Wang X, Wang Y, Lan H, Li J. MiR-195 inhibits the growth and metastasis of NSCLC cells by targeting IGF1R. *Tumour Biol* 2014, **35**(9)**:** 8765-8770.
